# Supplementary material for: Changes of Arbuscular Mycorrhizal Fungal Community and Glomalin in the Rhizosphere along the Distribution Gradient of Zonal Stipa Populations across the Arid and Semiarid Steppe
Source: Microbiol Spectr. 2022 Oct 10;10(5):e01489-22. doi: 10.1128/spectrum.01489-22 (PMC9602637; doi:10.1128/spectrum.01489-22)
Supplement: Supplemental file 1 — Supplemental material. Download spectrum.01489-22-s0001.pdf, PDF file, 1.8 MB [file spectrum.01489-22-s0001.pdf]

Supplementary Material

Table S1 Characteristics of the thirty-two study sites.

Stipa taxa	Sample	Sampling region	Grassland type	Latitude/longitude coordinates (D)	Altitude (m)	MAT (℃)	MAP (mm)
Sba1	S1	Ergun Banner	M	E119.6157 / N50.1194	525.9	1.15	442.35
	S2	Hailar	M	E120.2124 / N49.6320	673.9	-0.12	448.30
	S3	Yimin Sumu	M	E119.6691 / N48.4934	745	1.30	406.83
	S4	Xin Barag Left Banner	M	E118.9482 / N48.0936	761	1.62	362.97
	S5	East Ujimqin Banner	M	E119.3330 / N46.1402	987.7	1.04	472.06
	S6	East Ujimqin Banner	M	E119.5269 / N45.7584	930.4	2.24	452.74
Sgr2	S7	Prairie Chenbarhu banner	T	E118.8338 / N48.8809	717.5	1.53	336.83
	S8	Xin Barag Left Banner	T	E118.8734 / N48.1163	752	1.69	354.82
	S9	East Ujimqin Banner	T	E118.7262 / N46.3169	875.8	2.17	426.05
	S10	East Ujimqin Banner	T	E117.7865 / N45.9150	948.7	2.09	329.03
	S11	West Ujimqin banner	M	E118.8480 / N44.9533	984.9	2.12	402.51
	S12	West Ujimqin banner	M	E117.7260 / N44.4660	1163.2	3.15	406.26
	S13	East Ujimqin Banner	T	E116.6281 / N44.9774	851	3.98	320.84
	S14	xilin hot	T	E115.9876 / N44.4933	989.7	4.66	308.75
	S15	xilin hot	T	E116.4467 / N44.1004	1098	3.79	324.89
	S16	xilin hot	T	E116.1660 / N43.4287	1458.1	2.06	360.59
	S17	Zhenglan Banner	T	E116.5212 / N42.4600	1263	4.08	430.50
Skr3	S18	xilin hot	T	E115.9614 / N44.6105	1016.5	4.32	300.67
	S19	Abag Banner	T	E115.4065 / N43.7963	1125.6	4.23	302.11
	S20	xilin hot	T	E116.0853 / N43.5012	1378	3.06	340.00
	S21	Zhengxiangbai Banner	T	E114.7686 / N42.4920	1295.8	4.90	327.04
	S22	Chahar Right Back Banner	T	E113.5821 / N41.9165	1466	4.32	375.64
	S23	Abag Banner	T	E114.2866 / N43.3581	1081.5	5.37	234.39
	S24	Abag Banner	T	E114.3127 / N44.0816	1058	4.78	233.11
	S25	Sonid Left Banner	D	E113.0181 / N44.0591	980.3	5.59	172.19
Sgl7	S26	Erenhot	D	E112.2074 / N43.5742	960.5	6.25	152.08
Skl6	S27	Sonid Right Banner	D	E112.9513 / N42.9628	1076	6.56	220.81
Sbr4	S28	Sonid Right Banner	D	E112.8079 / N42.3102	1208	6.18	254.66
	S29	Siziwang Banner	D	E111.9378 / N41.7702	1439.3	4.98	331.14
	S30	Baotou	D	E110.6313 / N41.5279	1445	5.63	355.48
Stg5	S31	Baotou	D	E109.5182 / N41.9235	1498.5	5.50	248.98
	S32	Urad Front Banner	D	E108.4610 / N41.7851	1531	5.65	232.31

Notes: *S. baicalensis* (Sba1), *S. grandis* (Sgr2), *S. krylovii* (Skr3), *S. glareosa* (Sgl7), *S. klemenzii* (Skl6), *S. breviflora* (Sbr4), and *S. gobica* (Stg5). MAT: mean annual temperature; MAP: mean annual precipitation.; M: meadow steppe; T: typical steppe; D: desert steppe.

Table S2 *Stipa* taxa root-zone soil physicochemical, element and plant index contents of 32 sample sites.

Stipa taxa	Sample	pH	SOC g/kg	TN g/kg	AP μg/g	AK mg/kg	OP μmol/g	Pi μmol/g	TP μmol/g	Mg mg/L	Fe mg/L	Cu mg/L	Zn mg/L	Mn mg/L	S mg/L	Na mg/L	Ca mg/L	PH	VC %	SB g/per	EEG g/kg	TG g/kg	SM %
Sba1	S1	6.49±0.01	22.48±0.06	2.46±0.29	11.27±0.12	55.91±0.40	15.74±3.54	12.33±2.34	28.07±3.62	27.60±0.07	99.54±0.07	0.1245±0.08	0.2347±0.02	2.88±0.08	4.71±0.08	21.86±0.01	46.20±0.01	2.32±0.10	60.00±4.35	3.73±0.26	3.55±0.59	9.55±1.15	11.5±3.71
	S2	6.42±0.01	20.51±0.09	3.38±0.28	22.84±0.41	68.66±0.17	19.47±4.31	9.4±2.05	28.87±2.34	26.50±0.07	105.20±0.09	0.1239±0.05	0.2853±0.01	3.218±0.01	4.96±0.03	21.85±0.02	52.80±0.05	2.44±0.31	65.00±2.53	2.14±0.14	5.22±0.96	10.25±0.56	19.53±2.65
	S3	6.88±0.05	23.04±0.05	1.11±0.25	20.67±0.17	28.26±0.10	10.8±2.12	17.6±1.75	28.4±2.12	23.01±0.09	74.85±0.03	0.0858±0.01	0.1646±0.08	1.852±0.03	3.42±0.07	21.87±0.01	55.85±0.09	2.79±0.08	80.00±3.8	1.4±0.16	3.04±0.16	3.71±0.14	28.1±3.21
	S4	6.06±0.02	17.00±0.09	1.38±0.13	14.97±0.04	82.25±0.55	14±2.34	8.94±1.25	22.93±2.18	17.78±0.05	68.17±0.07	0.0989±0.02	0.167±0.05	1.963±0.09	3.12±0.01	21.83±0.03	41.37±0.00	1.64±0.05	40.00±2.54	2.13±0.13	5.18±0.09	6.85±0.34	2.77±2.15
	S5	6.64±0.03	15.72±0.07	1.54±0.15	10.71±0.20	49.46±0.04	14.91±1.65	11.51±1.36	26.42±1.58	39.61±0.08	119.40±0.05	0.1543±0.06	0.308±0.01	3.431±0.09	3.33±0.07	21.87±0.05	120.60±0.04	1.96±0.29	76.67±2.17	3.35±0.22	3.42±0.05	8.14±0.82	33.47±5.21
	S6	7.32±0.02	22.28±0.06	1.77±0.27	18.99±0.08	48.87±0.18	13.62±1.45	5.29±0.85	18.91±2.25	29.20±0.02	92.55±0.04	0.1301±0.06	0.2069±0.06	2.256±0.06	4.28±0.04	21.87±0.08	56.53±0.06	2.35±0.19	78.33±2.50	5.25±0.28	4.08±0.32	5.87±0.46	17.57±3.24
Sgr2	S7	6.49±0.03	16.00±0.78	2.70±0.15	19.62±0.17	68.03±0.44	16.93±3.21	11.07±1.35	28±2.36	28.98±0.04	98.96±0.01	0.1334±0.08	0.2175±0.05	2.723±0.09	0.81±0.08	21.85±0.06	47.70±0.04	1.18±0.44	40.00±2.65	1.67±0.15	4.65±0.05	5.55±1.06	15.93±4.21
	S8	6.26±0.03	13.00±0.05	2.26±0.26	21.21±0.13	77.21±0.67	12.94±3.36	17.73±1.47	30.67±3.45	20.62±0.01	76.55±0.01	0.1017±0.05	0.1714±0.01	2.211±0.03	3.30±0.01	21.85±0.02	40.62±0.08	2.24±0.27	43.33±3.54	0.58±0.26	2.49±0.34	4.51±0.88	3.13±2.13
	S9	6.65±0.04	8.55±0.02	0.62±0.12	18.60±0.01	69.08±0.57	11.86±0.92	11.47±1.25	23.33±2.15	10.90±0.04	40.76±0.08	0.0703±0.00	0.1191±0.07	1.013±0.05	2.82±0.07	21.86±0.03	27.82±0.08	1.85±0.02	60.00±2.74	3±0.12	2.82±0.3	3.89±0.33	10.27±3.25
	S10	6.46±0.01	8.78±0.51	0.80±0.27	15.80±0.04	80.59±1.19	18.4±1.68	9.53±1.36	27.93±2.21	14.05±0.01	54.91±0.08	0.0899±0.02	0.0445±0.03	1.282±0.05	1.80±0.00	20.98±0.08	29.24±0.02	1.67±0.09	40.00±2.24	3.02±0.25	1.88±0.03	2.51±0.21	16.4±4.25
	S11	6.53±0.05	10.26±0.12	1.25±0.25	17.68±0.06	81.97±0.75	6.27±2.34	18.22±2.35	24.49±1.12	18.55±0.05	69.07±0.06	0.0921±0.06	0.1584±0.06	1.806±0.00	3.10±0.06	21.86±0.06	33.40±0.09	1.82±0.39	65.00±2.74	3.2±0.31	1.52±0.35	2.74±0.25	11.5±3.26
	S12	6.88±0.03	12.02±0.42	2.18±0.36	11.35±0.15	81.72±0.60	10.47±2.51	9.8±1.86	20.27±1.98	15.34±0.01	60.03±0.05	0.0867±0.09	0.1132±0.04	1.532±0.03	2.15±0.00	21.89±0.01	29.03±0.09	2.16±0.62	66.67±0.91	2.97±0.24	2.69±0.36	4.33±0.33	22.57±3.24
	S13	7.44±0.05	7.23±0.27	0.97±0.06	21.35±0.16	69.59±0.79	9.47±2.46	8.47±1.57	17.93±1.95	33.28±0.01	99.74±0.07	0.1065±0.04	0.1667±0.09	2.904±0.06	1.17±0.07	20.99±0.06	38.74±0.07	1.76±0.14	36.67±1.49	3±0.27	0.93±0.18	2.16±0.23	8.87±3.25
	S14	7.29±0.05	8.00±0.14	0.80±0.10	21.59±0.13	60.73±1.22	12.34±0.58	8.13±1.26	18.97±2.34	24.57±0.01	78.25±0.05	0.0931±0.05	0.0981±0.07	2.248±0.08	2.45±0.07	21.01±0.03	129.00±0.06	1.61±0.29	30.00±2.36	3.76±0.14	2.24±0.1	3.48±0.23	8.63±3.14
	S15	8.18±0.05	17.08±0.12	1.92±0.15	24.03±0.24	57.8±0.37	5.27±0.67	12±1.11	17.27±2.39	22.48±0.06	79.99±0.06	0.1124±0.06	0.149±0.08	1.905±0.02	3.58±0.01	21.86±0.05	163.70±0.04	1.80±0.33	50.00±2.95	2.89±0.28	1.74±0.03	2.59±0.51	17.07±2.85
	S16	6.9±0.01	18.50±0.11	1.64±0.12	21.31±0.15	94.56±0.52	6.14±1.1	10.93±1.25	17.07±2.21	27.68±0.07	92.37±0.04	0.1243±0.05	0.2498±0.00	2.247±0.09	3.22±0.07	21.9±0.01	41.85±0.07	2.18±0.06	35.00±2.53	3±0.27	2.35±0.09	3.16±0.08	3.6±2.45
	S17	7.57±0.02	10.01±0.12	0.84±0.09	13.89±1.66	62.97±0.49	11.33±2.1	14±1.36	25.33±1.59	23.68±0.05	71.99±0.07	0.1043±0.08	0.1817±0.08	1.787±0.00	2.38±0.09	21.88±0.03	35.37±0.00	2.22±0.21	55.00±2.31	3.55±0.12	1.82±0.17	2.26±0.03	21.13±3.26
Skr3	S18	7.88±0.02	8.56±0.73	0.97±0.07	5.62±0.09	59.51±0.49	7.17±1.15	11.47±2.1	19.8±1.85	24.57±0.08	78.25±0.07	0.0931±0.04	0.0981±0.03	2.248±0.07	2.45±0.04	21.01±0.02	129.00±0.00	2.32±0.49	35.00±2.53	1.64±0.09	0.92±0.19	1.61±0.04	9.27±4.23
	S19	7.45±0.03	12.78±0.16	0.96±0.09	17.35±0.22	59.72±0.33	6.73±0.85	14.44±2.45	21.18±2.17	28.35±0.01	88.85±0.06	0.0989±0.01	0.1234±0.07	1.855±0.08	2.06±0.06	20.98±0.03	48.81±0.02	2.04±0.71	35.00±2.74	2.87±0.17	2.16±0.26	4.16±1.09	16.1±4.35
	S20	7.74±0.02	18.5±0.38	1.99±0.26	22.68±0.30	95.70±0.47	6.27±0.47	10.4±1.2	16.67±2.14	16.50±0.08	53.38±0.08	0.0823±0.07	0.1072±0.06	1.389±0.09	2.63±0.05	21.92±0.05	30.31±0.00	1.84±0.01	30.00±2.53	1.02±0.16	0.99±0.1	1.82±0.1	2.93±2.6
	S21	6.75±0.03	7.84±0.04	0.87±0.06	18.56±0.08	72.81±0.50	4.27±0.35	12.8±1.01	17.07±2.1	26.90±0.06	73.99±0.05	0.1107±0.08	0.2774±0.05	1.854±0.08	2.23±0.01	21.89±0.08	45.17±0.00	1.97±0.05	45.00±2.19	1.2±0.07	2.69±0.32	4.57±0.31	20.23±3.45
	S22	8.46±0.01	9.00±0.08	1.14±0.10	23.01±0.49	88.33±1.58	5.2±0.46	18±1.58	23.2±1.28	38.32±0.03	115.90±0.02	0.1355±0.07	0.3509±0.07	2.916±0.07	5.36±0.02	21.81±0.06	111.00±0.07	1.86±0.10	40.00±2.65	0.9±0.13	2.59±0.28	2.91±0.11	20.33±3.21
	S23	7.75±0.04	7.96±0.03	0.49±0.04	11.87±0.61	95.91±0.96	10.58±1.25	13.2±2.39	23.78±1.35	26.32±0.08	82.26±0.07	0.1015±0.08	0.1112±0.01	2.015±0.07	1.44±0.07	20.97±0.01	48.30±0.00	1.93±0.50	40.00±2.59	0.64±0.06	0.49±0.16	1.05±0.08	11.8±3.25
	S24	7.25±0.05	8.82±0.11	0.42±0.06	26.78±0.25	99.21±1.10	5.09±1.32	17.15±2.41	22.24±1.68	11.84±0.09	49.00±0.07	0.0558±0.04	0.0316±0.06	1.177±0.08	0.94±0.04	20.95±0.03	30.15±0.08	2.29±0.20	10.00±2.17	0.95±0.09	0.79±0.21	2.75±0.36	13.9±3.21
	S25	7.48±0.01	9.24±0.04	0.53±0.06	24.42±0.26	87.08±1.26	3.54±0.45	16.13±2.13	19.67±1.84	17.93±0.08	60.05±0.07	0.0679±0.07	0.0412±0.07	1.285±0.08	1.15±0.08	20.95±0.02	26.62±0.02	1.94±0.36	20.00±2.21	0.74±0.06	0.42±0.04	3.03±2.67	14.13±3.58
Sgl7	S26	7.99±0.05	4.94±0.12	0.17±0.02	22.23±0.16	77.41±0.70	4.36±0.56	8.84±0.95	13.2±1.69	8.57±0.05	29.89±0.07	0.062±0.00	0.0132±0.09	1.388±0.04	0.92±0.08	20.99±0.03	18.57±0.06	2.26±0.46	14.00±2.16	0.96±0.11	0.14±0.08	1.01±0.1	3.07±2.65

Skl6	S27	8.21±0.03	5.50±0.40	0.22±0.04	25.58±0.42	69.85±0.75	6.67±0.42	17.24±2.36	23.91±2.14	17.97±0.02	54.32±0.05	0.078±0.04	0.0766±0.07	1.187±0.08	1.30±0.03	20.94±0.05	42.41±0.08	1.74±0.39	10.00±2.07	0.72±0.09	0.59±0.08	1.45±0.04	5.87±2.31
	S28	7.95±0.09	4.76±0.06	0.80±0.08	23.84±0.51	77.46±0.59	4.54±0.35	10.93±1.24	15.47±1.02	36.98±0.04	104.60±0.05	0.1423±0.07	0.1688±0.07	2.637±0.05	2.08±0.02	20.98±0.01	55.25±0.05	2.05±0.33	23.33±2.06	0.84±0.02	1.54±0.1	2.86±0.24	15.17±2.85
Sbr4	S29	8.07±0.02	10.42±0.53	0.39±0.01	30.15±0.32	81.15±1.48	3.87±0.25	16±1.58	19.87±1.8	37.52±0.07	106.40±0.07	0.123±0.06	0.2332±0.07	2.807±0.05	3.39±0.07	20.98±0.08	149.40±0.04	2.11±0.11	31.67±2.24	1.22±0.12	1.76±0.3	3.15±0.3	5.83±2.35
	S30	7.92±0.03	6.02±0.12	0.25±0.01	30.40±0.18	49.96±0.29	5.8±0.36	14.07±1.95	19.87±0.98	27.96±0.08	83.39±0.04	0.095±0.07	0.0968±0.09	2.14±0.01	1.42±0.04	20.94±0.06	60.04±0.04	2.02±0.84	27.50±3.61	0.49±0.05	0.84±0.23	2.14±0.4	13.47±6.54
Stg5	S31	7.88±0.02	7.06±0.46	0.31±0.04	28.03±0.25	61.87±0.55	2.47±0.1	14.53±2.31	17±2.65	34.36±0.09	101.80±0.06	0.1127±0.06	0.1598±0.05	2.313±0.05	1.59±0.00	20.92±0.01	66.10±0.05	1.32±0.99	16.67±0.91	0.29±0.07	0.99±0	2.71±0.52	5.97±3.21
	S32	8.74±0.02	5.54±0.03	0.27±0.03	21.75±0.52	64.45±0.64	2.57±0.15	13.67±1.85	16.23±2.45	42.81±0.06	115.80±0.06	0.1511±0.08	0.3342±0.09	2.963±0.02	2.00±0.05	21.82±0.05	62.95±0.01	0.84±0.57	16.67±2.04	0.31±0.03	1.62±0.26	3.04±0.35	4.3±2.14

Notes SOC: soil organic carbon; TN: total nitrogen; AP: alkaline neutral available phosphorus; AK: available potassium; Fe: iron element; Cu: copper element; Zn: zinc element; Mn:manganese element; Ca: calcium element; S: sulfur element; Mg: Magnesium element; VS： Shannon-Wiener index of plot; VC: Vegetation coverage; SB: Biomass of Stipa, SM: Soil Moisture; OP: Organic Phosphorus; Pi: Inorganic Phosphorus; TP: Total Phosphorus; TG: Total Glomalin; EEG: Easily-Extractable Glomalin; long: longitude; lat: latitude. *S. baicalensis* (Sba1), *S. grandis* (Sgr2), *S. krylovii* (Skr3), *S. breviflora* (Sbr4), *S. gobica* (Stg5), *S. klemenzii* (Skl6) and *S. glareosa* (Sgl7).

Table S3 Sequence characteristics obtained in the root-zone soil of *Stipa* taxa. In the table the raw read and after processing read counts are presented as well as three α diversity indices.

Sample\Info	Mean raw reads	sd	Sequences	sd	Base_num	sd	Mean_length	sd	OUT number	sd	Shannon	sd	Chao1	sd	Pielou	sd
S1	24262	390	23986	343	5256436	97867	217	1	109	16	5.14	0.25	64	14.73	0.86	0.05
S2	24001	114	23652	124	5185239	25914	216	0	86	18	4.31	0.28	35.33	10.41	0.84	0.03
S3	24379	487	23902	773	5292306	117159	217	1	82	25	4.35	0.35	33.67	11.59	0.87	0.03
S4	22850	1690	21953	1601	4929268	366025	216	0	182	16	5.60	0.09	94	8.89	0.85	0
S5	23867	774	23554	747	5169009	174960	217	1	84	13	4.58	0.24	37.67	5.51	0.88	0.06
S6	23850	684	23517	587	5153965	145856	216	0	105	10	5.04	0.05	51	7.94	0.89	0.01
S7	23822	152	23595	195	5156796	38620	216	1	118	12	5.40	0.14	69.67	17.01	0.89	0.01
S8	24199	696	23120	437	5217661	147974	216	0	239	108	5.92	0.28	138.33	70.87	0.85	0.03
S9	24236	168	24013	217	5249564	56565	216	2	193	111	5.22	0.71	117.33	80.96	0.81	0.05
S10	23076	986	22801	1044	4987064	207401	216	0	274	28	6.00	0.25	181.67	51.05	0.8	0.03
S11	22297	2143	22090	2105	4813961	452139	216	1	203	131	5.33	0.62	117.67	90.72	0.82	0.05
S12	20942	2496	20675	2510	4546646	548771	217	1	68	14	3.66	0.38	31	3.61	0.74	0.13
S13	24080	764	23817	708	5187251	163951	215	0	121	6	5.17	0.25	79.33	10.6	0.82	0.04
S14	22098	3787	21827	3800	4787312	811316	217	1	201	171	5.69	0.76	143	149.23	0.86	0.06
S15	23930	657	23724	667	5178746	135876	216	1	102	5	5.28	0.05	58.33	5.03	0.9	0.02
S16	23574	405	22806	445	5079474	90592	215	1	163	2	5.57	0.05	102	6.24	0.84	0.01
S17	23576	677	23148	906	5095375	144067	216	0	258	104	6.18	0.24	180.33	61.65	0.84	0.09
S18	22296	2192	22092	2150	4812301	472527	216	0	53	22	3.50	0.54	17.67	9.29	0.87	0.05
S19	23820	704	23621	675	5133925	148813	215	1	88	23	4.81	0.3	54.67	21.94	0.85	0.03
S20	23962	500	23137	968	5169080	109568	216	0	232	72	5.90	0.09	144.33	55.19	0.83	0.07
S21	24361	548	23897	443	5256841	115989	216	0	125	6	5.54	0.22	80.33	6.11	0.88	0.04
S22	24297	494	23733	380	5247097	106745	216	0	159	9	5.74	0.12	97.67	9.81	0.87	0.01
S23	23604	472	23484	457	5086599	99003	216	1	58	34	3.22	0.31	22.33	11.59	0.76	0.08
S24	23734	1237	23457	1192	5180111	266888	218	1	45	5	3.63	0.08	16.67	3.06	0.9	0.07
S25	22368	2196	22084	2207	4841783	454818	217	1	44	12	3.07	0.42	13.67	5.86	0.84	0.06
S26	23715	839	23449	843	5121525	179766	216	0	37	14	2.74	0.51	9.33	3.79	0.87	0.09
S27	22973	427	22500	454	4955936	92526	216	0	87	4	4.47	0.12	34	6.56	0.88	0.02
S28	11963	1297	11339	1215	2582614	281070	216	0	194	2	5.59	0.13	80.33	4.51	0.88	0.02
S29	13246	1879	12647	1896	2862838	405387	216	1	75	25	3.74	0.51	18	6.56	0.91	0.06
S30	15128	882	14632	604	3289360	199996	217	2	58	14	3.27	0.1	16	5	0.83	0.04
S31	15195	4271	14651	4169	3274302	920027	215	1	156	10	5.12	0.14	65	3.61	0.85	0.03
S32	24037	452	23582	492	5198262	100655	216	0	180	8	5.62	0.04	105.33	4.73	0.84	0.01
mean	22304		21890		4821833		216		131		4.82		72.18		0.85	
Total	2141212		2101443		462895944				12541							

Table S4 The distribution and relative abundance of AMF order, family and genus of AMF species among root-zone of seven *Stipa* species from 32 sites. O: Order, F: Family, G: Genus.

Order	Family	Genus	Species (numbers)	Sba1 (%)			Sgr2 (%)			Skr3 (%)			Sbr4 (%)			Stg 5(%)			Skl 6(%)			Sgl7 (%)		
				O	F	G	O	F	G	O	F	G	O	F	G	O	F	G	O	F	G	O	F	G
	Acaulosporaceae	*Acaulospora*	1		-	-		-	-		-	-		-	-		-	-		-	-		-	-
		Diversispora	4			4.67			5.67			0.1			-			0.04			0.25			-
Diversisporales	*Diversisporaceae*	*unclassified_f__Diversisporaceae*	1	5.85	4.84	-	7.97	6.38	-	0.11	0.1	-	-	-	-	0.04	0.04	-	0.25	0.25	-	-	-	-
		Gigaspora	1			-			-			-			-			-			-			-
	Gigasporaceae	*Scutellospora*	3		1.01	1.01		1.57	1.57		-	0.01		-	-		-	-		-	-		-	-
	Ambisporaceae	*Ambispora*	1		-	-		-	-		-	-		-	-		-	-		-	-		-	-
Archaeosporales	*Archaeosporaceae*	*Archaeospora*	2	0.97	-	-	0.58	-	-	-	-	-	-	-	-	-	-	-	-	-	-	-	-	-
	unclassified_o__Archaeosporales	*unclassified_o__Archaeosporales*	1		-	-		-	-		-	-		-	-		-	-		-	-		-	-
Glomerales	*Glomeraceae*	*Glomus_f__Glomeraceae*	61	86.06	86.06	86.06	84.88	84.88	84.88	96.47	96.47	96.47	92.49	92.49	92.49	99.72	99.72	99.72	94.16	94.16	94.16	93.21	93.21	93.21
Paraglomerales	*Paraglomeraceae*	*Paraglomus*	7	5.94	5.94	5.94	1.68	1.68	1.6	0.34	0.34	0.34	1.8	1.8	1.8	-	-	-	-	-	-	-	-	-
unclassified_c__Glomeromycetes	*unclassified_c__Glomeromycetes*	*unclassified_c__Glomeromycetes*	1	1.18	1.18	1.18	4.89	4.89	4.89	3.07	3.07	3.07	5.71	5.71	5.71	0.24	0.24	0.24	5.59	5.59	5.59	6.79	6.79	6.79

S. baicalensis (Sba1), *S. grandis* (Sgr2), *S. krylovii* (Skr3), *S. breviflora* (Sbr4), *S. gobica* (Stg5), *S. klemenzii* (Skl6) and *S. glareosa* (Sgl7).

Table S5 Influence of ecological environmental factors on AMF diversity. *Correlation is significant at the 0.05 level.**Correlation is significant at the 0.01 level.

Index	AK	pH	SOC	TN	AP	OP	Pi	TP	SM	TG	EEG	Long	Lat	*Stipa* type
**Shannon**	-0.02	**-.212***	0.176	**.266****	-0.06	**.216***	-0.099	0.137	-0.05	0.151	**.341****	**.253***	0.111	-0.18
**Chao1**	0.118	-0.198	-0.005	0.096	-0.108	**.207***	-0.087	0.135	-0.13	-0.051	0.113	0.188	0.033	-0.16
**Pielou**	-0.177	0.13	0.096	-0.016	**.248***	-0.117	0.08	-0.065	0.015	0.094	0.088	-0.063	-0.001	0.081
	**MAP**	**MAT**	**SB**	**PH**	**VC**	**S**	**Ca**	**Fe**	**Cu**	**Zn**	**Mn**	**Mg**	**Alt**	
**Shannon**	**.348****	**-.316****	**.288****	**-.339****	0.159	**.302****	-0.007	**.221***	**.378****	**.399****	0.16	0.196	0.041	
**Chao1**	**.227***	**-.214***	**.239***	**-.266****	0.075	0.122	-0.135	-0.054	0.082	0.11	-0.072	-0.056	0.052	
**Pielou**	-0.057	0.063	-0.048	0.053	-0.113	0.119	**.314****	0.152	0.155	0.135	0.158	0.17	-0.011	

Table S6 Statistical output of PERMANOVA (Adonis, vegan package) testing the effect of *Stipa* population on the AMF composition (999 permutations, Bonferroni adjusted *P* values).

Characteristics	Df	SumsOfSqs	MeanSqs	F_Model	R^2	*P*_value	*P*_adjust
Stipa	7	2.75948575	0.459914292	3.9047876	0.208387363	0.001	0.001
Residuals	89	10.48261164	0.117782153	-	0.791612637	-	
Total	96	13.24209739	-	-	1	-	

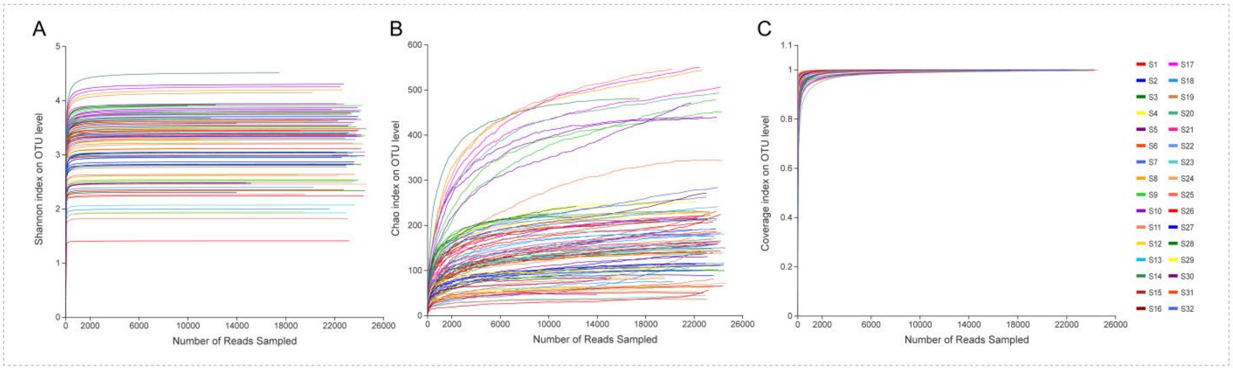

FIG S1 The rarefaction curve analysis of the AMF sequences. The curves were constructed using the Shannon (A) Chao (B) and Coverage (C) index values of the OTUs.

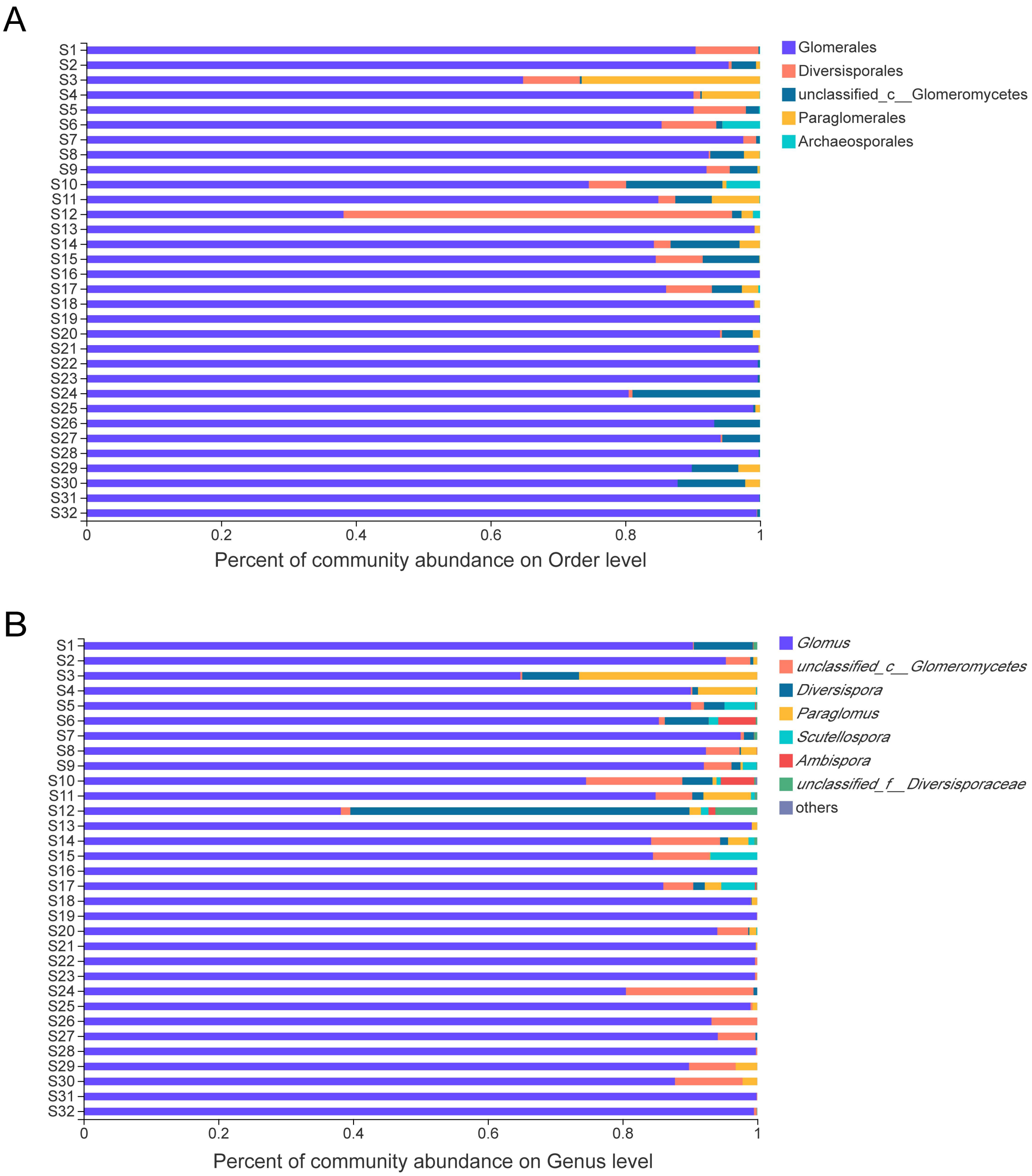

FIG S2 AMF community structure in *Stipa* root zone at different taxonomic levels in 32 sites. a) The percent of community abundance aon order level. b) the percent of community abundance on genus level.

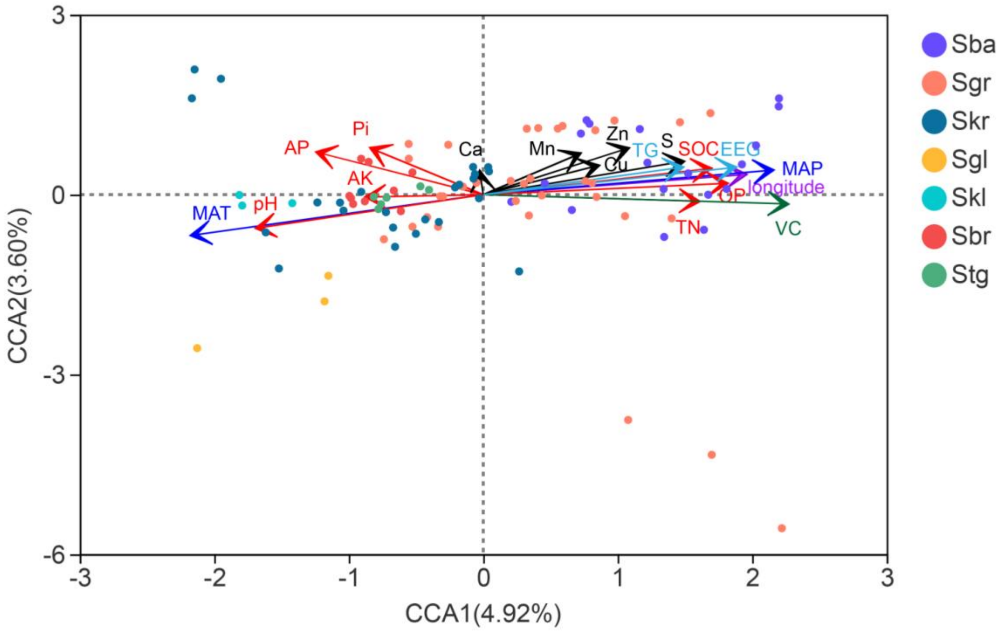

FIG S3 Relationships between AMF community structure and environmental factors used canonical correlations analysis (CCA). Differently colored arrows correspond to different types of variables. Notes SOC: soil organic carbon; TN: total nitrogen; AP: alkaline neutral available phosphorus; AK: available potassium; Cu: copper element; Zn: zinc element; Mn: manganese element; Ca: calcium element; S: sulfur element; VC: Vegetation coverage; OP: Organic Phosphorus; Pi: Inorganic Phosphorus; TG: Total Glomalin; EEG: Easily-Extractable Glomalin; long: longitude.
